# Mechanism of a Herbal Formula Associated with Prognosis and Immune Infiltration in LIHC: Transcriptomics Analysis and Molecular Dynamics Simulations

**DOI:** 10.1155/2022/6084321

**Published:** 2022-06-15

**Authors:** Jiankun Xu, Defu Liu, Zhanhua Liu, Jing Yang, Hanrui Chen

**Affiliations:** ^1^The First Clinical Medical College of Guangzhou University of Chinese Medicine, Guangzhou 510405, China; ^2^The Basic Medical College of Guangzhou University of Chinese Medicine, Guangzhou 510405, China; ^3^The First Affiliated Hospital of Guangzhou University of Chinese Medicine, Guangzhou 510405, China; ^4^The Pharmaceutical College of Guangzhou University of Chinese Medicine, Guangzhou 510405, China

## Abstract

**Background:**

The aim of this study is to explore the interactions between effective monomers of herbal formulas and their therapeutic targets using systems biology approaches which may be a promising approach to unraveling their underlying mechanisms. Shentao Ruangan decoction (STRGD), which has been experimentally, clinically demonstrated to be effective in treating liver hepatocellular carcinoma (LIHC), was selected.

**Methods:**

Bioactive ingredients and drug targets of STRGD were retrieved from the traditional Chinese medicine systems pharmacology database and analysis platform and BATMAN-TCM databases. LIHC-related differentially expressed genes (DEGs) and key modules were identified by a weighted gene coexpression network analysis using The Cancer Genome Atlas data. The Kaplan–Meier analysis was used to investigate the relationship between STRGD tumor targets and patients survival. The CIBERSORT deconvolution algorithm was used to analyze the correlation between STRGD tumor targets and infiltrating immune cells. Enrichment analysis was used to analyze biological functions. Interactions between STRGD compounds and LIHC-immune-related genes were investigated using molecular docking and MDS.

**Results:**

We identified 24 STRGD tumor targets, which were found to be correlated with survival and the level of immune cell infiltration in LIHC patients. Immune infiltration, gene set enrichment, and Kyoto Encyclopedia of Genes and Genomes analyses highlighted the roles of T and B cell subsets, which were both related to activator protein 1 (AP1), in STRGD action. Docking studies and HPLC indicated that tanshinone IIA is the main compound of STRGD in LIHC treatment, and MDS showed that the potential LIHC-immune-related targets 1FOS and 1JUN firmly bind to tanshinone IIA.

**Conclusions:**

The mechanisms of STRGD in improving the immune and survival status of LIHC patients include interactions between STRGD compounds and LIHC-immune-related targets. The findings of this study can guide research studies on the potential usefulness of tanshinone IIA in the development of drugs targeting 1JUN and 1FOS for the treatment of LIHC.

## 1. Background

Primary liver cancer, a malignant tumor associated with high morbidity and mortality, includes pathological types such as liver hepatocellular carcinoma (LIHC), intrahepatic cholangiocarcinoma (ICC), and mixed hepatocellular carcinoma [1]. LIHC accounts for approximately 90% of liver cancers [2]. The International Agency for Research on Cancer of the World Health Organization reported that liver cancer was the sixth most common cancer worldwide in 2020 [3]. Furthermore, liver cancer ranks in the top three in terms of mortality rate among all tumors and is the second most common cancer in men [3]. The disease prognosis is poor and with a 5-year survival rate of less than 10%, it is one of the cancers with the worst prognosis [4].

Treatment strategies for liver cancer include surgery, transplantation, transcatheter arterial chemoembolization (TACE), local ablation, chemotherapy, targeted therapy, immunotherapy, and traditional Chinese medicine (TCM). For small nodules of early liver cancer, surgical resection, transplantation, and local ablation are treatment options, whereas TACE, chemotherapy, targeted therapy, and immunotherapy are palliative treatments for advanced forms [5]. TCM is a complex medical system involving the use of multiple compounds that interact with multiple targets. TCM plays an indispensable role as a complementary and alternative therapy for end-stage liver disease, such as cirrhosis or liver cancer [6]. The effectiveness and safety of oral TCM preparations in the treatment of liver cancer has been confirmed [7]. Co-administration of oral and intravenous TCM preparations can prevent recurrence or metastasis of liver cancer after surgery, reduce adverse reactions of TACE, and improve the overall efficacy of TACE [8]. Compared with western medicine, TCM therapies for liver cancer have the advantages of being less costly and having fewer adverse reactions. As a symptomatic and supportive treatment, TCM can alleviate the effects of primary causative factors of early liver cancer, such as biological carcinogens and endocrine disorders, by enhancing tumor immunity [9, 10]. As such, TCM improves the survival rate, quality of life, and clinical benefit rate in patients with advanced liver cancer [9, 10].

Chinese herbal medicines, such as *Rheum palmatum*, *Angelica sinensis*, *Salvia miltiorrhiza*, Semen Persicae, *Panax quinquefolius*, *Agrimonia pilosa*, *Artemisia capillaris*, and Eupolyphaga Seu Steleophaga, a ground beetle preparation, have specific effects in the treatment of liver cancer. For example, *R. palmatum* blocked the cell cycle of hepatoma SMMC-7721 cells, whereas *A. capillaris* induced apoptosis of HepG2 cells [11]. *S. miltiorrhiza* inhibited the proliferation and invasion of hepatoma cells and tumor growth and metastasis [7, 12]. *P. quinquefolius* inhibited tumor growth in H22 tumor-bearing mice by regulating immunity [13]. Amygdalin in the Semen Persicae extract induced follistatin expression in HepG2 and C2C12 cells and the extract may be useful to treat liver fibrosis [14]. *A. sinensis* polysaccharide nanoparticles have been developed as a drug delivery system for liver cancer treatment because of their advantages of nontoxicity, low cost, good biocompatibility, and inherent liver targeting [15]. *A. pilosa* inhibited the proliferation of HepG2 cells [16]. Eupolyphaga Seu Steleophaga inhibited tumor growth in H22 tumor-bearing mice and induced apoptosis of hepatoma cells by regulating immunity and activating caspase-3 [17].

Shentao Ruangan decoction (STRGD) is a herbal formula prepared from a mixture of *R. palmatum*, *A. sinensis*, *S. miltiorrhiza*, Semen Persicae, *P. quinquefolius*, *A. pilosa*, *A. capillaris*, and Eupolyphaga Seu Steleophaga. Long-term clinical and experimental evidence shows that STRGD is effective in the treatment of LIHC. However, the identification of active compounds and antitumor targets remains unclear, and its pharmacological mechanisms have not been fully elucidated. STRGD inhibited the proliferation of BEL-7402 and SMMC-7721 hepatoma cells and synergistically improved the tumor-suppressive activity of lymphokine-activated killer cells [18]. In H22 tumor-bearing mice, STRGD inhibited tumor growth, enhanced interleukin-2 and natural killer cell activity, and restored and improved suppressed immune function [19]. STRGD has been shown to slow down progression of liver fibrosis, reduce damage to hepatocytes, and improve liver dysfunction [20], and to inhibit cell proliferation, induce apoptosis, and block the cell cycle in HepG2 cells [21]. In H22 tumor-bearing mice, STRGD inhibited telomerase activity and reduced the number of CD4+CD25+ regulatory T cells to inhibit tumor growth and increase body weight, thus improving the general condition of the mice [22].

In a clinical study, STRGD combined with hydroxycamptothecin for local interventional therapy of inoperable large liver cancer improved the Child-Pugh grade and indocyanine green results of patients [23]. Furthermore, this combination prolonged survival by protecting liver function and inhibiting tumor progression [23]. STRGD improved the clinical symptoms, Child-Pugh grade, and indocyanine green results of patients with recurrent liver cancer after surgery, stabilized the tumor, improved physical function, and prolonged progression-free and median survival [24].

The establishment and development of systems biology in recent years have promoted research on the systemic mechanisms of action and active ingredients of TCM preparations [7]. This development is conducive to the elucidation of TCM compounds and principles of formulated prescriptions based on TCM theory [7]. Data integration is the strength of systems biology, which allows analyzing individual entity data on different scales to translate the data into biological knowledge. Systems biology mainly uses experimental and computational tools combined with bioinformatics, network analysis, and statistical methods to analyze omics and high-throughput experimental data [25]. Herbal formulas with polyvalent components act on multiple targets in living organisms, and their complex biological effects depend on synergism of the active components. In contrast to conventional studies, such as clinical trials and generic treatment studies, which are reductionistic in nature, systems biology provides a holistic approach to studying biological systems and can reveal complex perturbations induced by herbal formulas [26]. Systems biology is beneficial for studying the network effects of polypharmacological approaches, which are applied in drug discovery.

**Figure 1:**
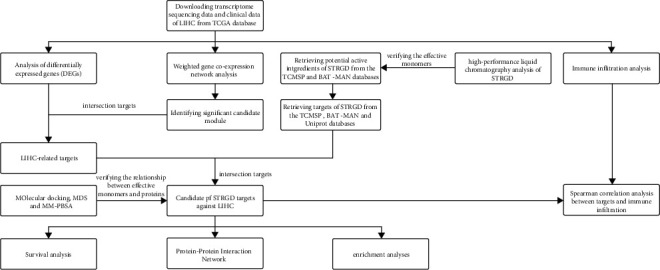
Flowchart of the study.

In this study, a top-down multiscale system research method was used. Protein-protein interaction (PPI) and weighted gene coexpression network analysis (WGCNA) topological networks were generated using high-dimensional omics or high-throughput sequencing data. Kaplan–Meier analysis, immune infiltration data, bioinformatics analyses, and a drug-compound-target-disease interactive network built by PPI network analysis were used to explore the possible pharmacological mechanisms of action of STRGD in LIHC. High-performance liquid chromatography (HPLC) and molecular docking were used for verification. Molecular dynamics simulation (MDS) provided clues for understanding the dynamic binding of lead compounds to core drug targets. This strategy allowed identifying the core drug targets of STRGD and exploring the potential interactions between lead compounds and LIHC. We expected the analyses to provide a theoretical basis for future experiments and clinical trials (Figure 1).

## 2. Methods

### 2.1. Collection of Data on the Chemical Ingredients and Targets of STRGD

Data on the chemical ingredients and targets of STRGD (*R. palmatum*, *A. sinensis*, *S. miltiorrhiza*, Semen Persicae, *P. quinquefolius*, *A. pilosa*, *A. capillaris*, and Eupolyphaga Seu Steleophaga) were retrieved from the TCM Systems Pharmacology Database and Analysis Platform (TCMSP) (https://tcmspw.com/index.php) [27] and BATMAN-TCM (http://bionet.ncpsb.org/batman-tcm/) databases [28]. To identify the potential active ingredients of STRGD, the criteria used for TCMSP database screening were standard oral bioavailability ≥ 30% and drug-like property ≥ 0.18. The screening criterion of the BAT-MAN database was score cutoff ≥ 20 [29]. The gene names of bioactive targets were then identified in the UniProt database.

### 2.2. STRGD Preparation

The STRGD ingredients were purchased from the First Affiliated Hospital of Guangzhou University of Chinese Medicine. The ingredients were soaked in deionized water (1 L) for 30 min, boiled for 40 min, and then simmered for 1 h. The resulting extract was concentrated at 100°C, filtered, centrifuged, and dried by lyophilization.

### 2.3. HPLC

HPLC is a chromatographic technique for the separation of multicomponent samples that can process compounds of different molecular weights and polarities. It is widely used for the identification, separation, and purification of chemical components in herbal formulas [30]. The sample solutions were injected into the HPLC system (Agilent 6540 Q-TOF and Agilent 1290 UHPLC, Agilent, Santa Clara, CA, USA) and separated using an ultrahigh performance-LC HSS T3 column (2.1 mm × 100 mm, 1.8 *μ*m, Elite, DaLian, LiaoNing, CN). The mobile phase consisted of 100% ultrapure water, 0.1% methanol (a), and 100% acetonitrile (b). The gradient elution program was as follows: 2% *B* at 0–1 min, 2%–100% *B* at 1–55 min, 100% *B* at 55–60 min, and 100%–2% *B* at 60–61 min. The flow rate was 0.4 mL/min, the injection volume was 10 *μ*L, and the column temperature was maintained at 50°C.

### 2.4. Acquisition of Targets Associated with LIHC

Transcriptome sequencing data, overall survival time, survival status, and other clinical information of patients with LIHC were retrieved from The Cancer Genome Atlas (TCGA) database (https://portal.gdc.cancer.gov/) [31]. The Limma package [32] was used for normalization, Log2 conversion, and differential gene expression analysis of the gene expression data. Differentially expressed genes (DEGs) were screened out based on |log2FC | > 1  and *P* < 0.05. WGCNA provides a network-based data reduction approach that uses unsupervised clustering to screen for gene modules closely related to the characteristics of disease samples. Moreover, in this technique, the gene modules are visualized as a hierarchical clustering dendrogram or heatmap plot of eigengenes [33]. The WGCNA *R* package was used to construct a coexpression network of all genes in the data set. Based on the scale-free network principle, the optimal soft threshold power *β* of WGCNA was selected from a threshold range of 1–20. The proximity matrix was built and converted into a topological overlap matrix. Based on the matrix, dissimilarity hierarchical clustering analysis was conducted by dissimilarity, and modules were delineated using the dynamic tree cut method. The minimum number of genes in each gene network module was set to 50 and the merge height was set to 0.25 to merge modules with similar feature vectors. Optimal modules were screened out according to the expression difference between LIHC and normal tissues (correlation coefficient [*R*] > 0.8, *P* < 0.01) [34]. All genes in the WGCNA optimal module were selected and intersected with the DEGs to identify disease targets. Targets at the intersection of disease targets and compound targets of STRGD were identified as tumor targets of STRGD. Disease targets and tumor targets of STRGD were mapped in Venn diagrams.

### 2.5. PPI Network Construction

PPI analysis contributes to enhancing the understanding of biological functions of intracellular proteins and identifying functional protein networks, potential biomarkers, and therapeutic targets [35]. STRING uses a specific algorithm to predict functional associations of targets and uses the estimated confidence score as a measure for building networks or filtering interactions [36]. The disease targets were uploaded to STRING (https://www.string-db.org/) and the minimum confidence score was set to ≥ 0.700. The PPI network analysis results and tumor targets of STRGD were imported into the Cytoscape 3.8.0 software to construct a drug-compound-target-disease network.

### 2.6. Identification of Survival-Related Targets

To find survival-related targets among the shared targets between drug and disease targets, the Survminer and Survival *R* packages were used to construct Kaplan–Meier survival curves and implement log-rank tests.

### 2.7. Biological Functional Analysis

Gene set enrichment analysis (GSEA) is a statistical method that can be used to determine whether a predefined gene set is differentially expressed in different phenotypes and to identify the biological processes represented by the gene set [37]. LIHC and normal tissues were used for phenotypic grouping, and the “c2. cp. Kegg. V7.4. symbols. gmt” cured gene set from Molecular Signatures Database was used as a reference gene set. The GSEA software was used to perform Kyoto Encyclopedia of Genes and Genomes (KEGG) pathway enrichment analysis of the LIHC transcriptome sequencing data, and the number of permutations was set to 1000. Using |normalized enrichment score| > 1, nominal *P* < 0.05, and false discovery rate (*q*) < 0.25 as criteria, cellular pathways significantly, differentially expressed between LIHC and normal tissues were screened out. Gene Ontology (GO) and KEGG pathway enrichment analyses were conducted on tumor targets of STRGD using the Colorspace, Stringi, DOSE, ClusterProfiler, and Pathview *R* packages, with *P* < 0.05 and *Q* < 0.05. For some key functions and pathways, the enrichment analysis results were visualized using bar diagrams and tree maps.

### 2.8. Immune Infiltration Analysis

CIBERSORT, an *in silico* approach to characterizing the composition of cell subsets in tissues based on gene expression profiles, can accurately estimate the immune cell composition in tumor tissues [38]. The CIBERSORT deconvolution algorithm was used to calculate the relative proportions of 22 types of tumor-infiltrating immune cells based on gene transcripts from LIHC and normal tissues. The screening criterion for samples was *P* < 0.05, and the number of naïve CD4+ T cells in all samples was 0. The distribution of the 22 immune cell types was visualized using the bar chart and box plot in ggplot2 R package. Using the immune cell correlation matrix combined with corresponding clinical data and the median immune cell proportions as a threshold value, the patients were divided into high-and low-infiltration groups. Survival analysis was conducted using the Survival and Survminer R packages, and Kaplan–Meier survival curves and log-rank tests were used to evaluate the correlation between immune cell infiltration patterns and LIHC prognosis. Spearman correlation analysis and the ggplot2 R package were used to construct a correlation heatmap to analyze the correlation between tumor targets of STRGD and immune cells.

### 2.9. Molecular Docking

Molecular docking was performed for genes that showed significant expression differences in the STRGD target-related survival analysis. Three-dimensional crystal structures of core target proteins were retrieved from the Research Collaboratory for Structural Bioinformatics Protein Data Bank (RCSB PDB, https://www.rcsb.org/) [39] and AlphaFold protein structure databases (https://alphafold.ebi.ac.uk/). The PyMOL software was used to remove water molecules, ions, heteroatoms, original ligands, and phosphate from the core target proteins. AutoDock Tools was used to add hydrogen, calculate total molecular charges, and save receptor files in the PDB Partial Charge (*Q*) and Atom Type (*T*) (PDBQT) format. The mol2 format structures of corresponding chemical compounds were retrieved from the TCMSP and BATMAN-TCM databases. We used AutoDock Tools to set the rotatable bonds and save the files of ligands in the PDBQT format. We used AutoDock-Vina for molecular docking and PyMol to analyze and visualize the docking results.

### 2.10. MDS and Molecular Mechanics Poisson–Boltzmann Surface Area (MM-PBSA) Calculations

MDS was performed using the Gromacs 5.14 software package [40], which is a collection of programs and libraries suitable for all types of MDS based on pair potentials. MDSs of the solvated and equilibrated protein-ligand complexes were conducted for 50 ns, and the 50-ns trajectories were analyzed using the MM-PBSA approach [41]. MM-PBSA is a computational method to quantify the strength of biomolecular interactions and evaluate the structural stabilities of docked complexes. Details of the MDS and MM-PBSA calculations are provided in the supplementary file.

## 3. Results

### 3.1. Acquisition of Active Compounds and Targets of STRGD

**Figure 2:**
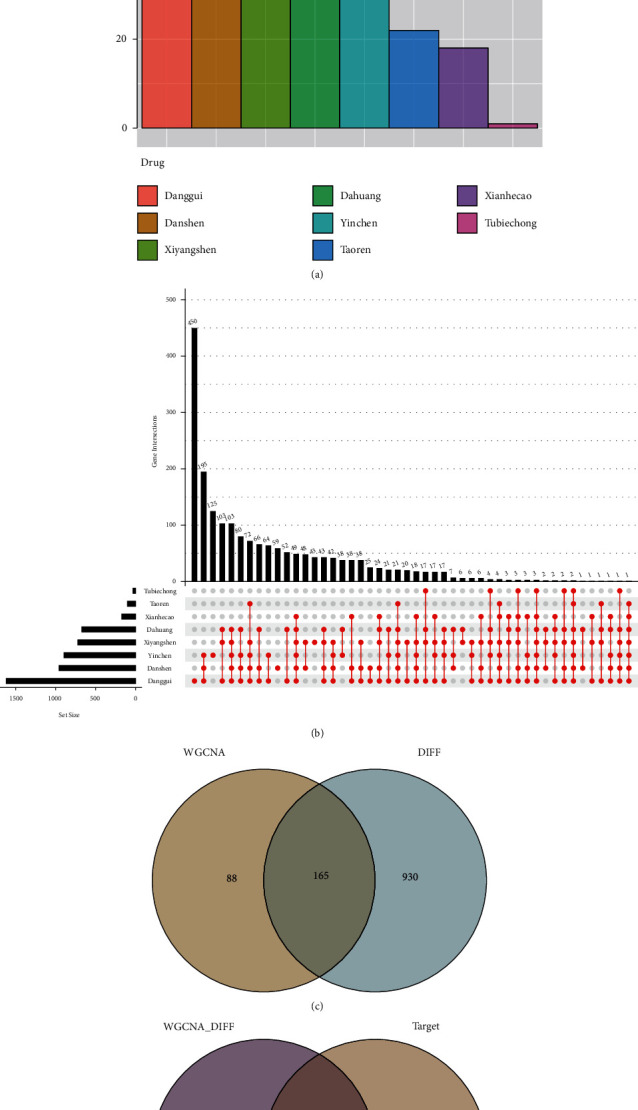
Diagram of compounds and targets in STRGD against LIHC. (a) Number of compounds of 8 herbs from STRGD. (b) Number of targets of 8 herbs from STRGD. (c) Venn diagram of targets related to LIHC. (d) Venn diagram of drug-disease intersection targets. The 165 targets of LIHC are mapped to the 2045 targets of STRGD to screen out the 24 common targets.

Based on the criteria of standard oral bioavailability ≥ 30%, drug-like property ≥ 0.18, and score cutoff ≥ 20, the active components of *R. palmatum*, *A. sinensis*, *S. miltiorrhiza*, Semen Persicae, *P. quinquefolius*, *A. pilosa*, *A. capillaris*, and Eupolyphaga Seu Steleophaga were retrieved from the TCMSP and BATMAN-TCM databases. A total of 148 active compounds were identified in STRGD, including 37, 84, 75, 22, 55, 18, 30, and 1 in *R. palmatum*, *A. sinensis*, *S. miltiorrhiza*, Semen Persicae, *P. quinquefolius*, *A. pilosa*, *A. capillaris*, and Eupolyphaga Seu Steleophaga, respectively (Figure 2(a)). We retrieved 2045 drug targets of the active compounds from the UniProt database, including 673, 1618, 957, 101, 722, 173, 896, and 32 targets for *R. palmatum*, *A. sinensis*, *S. miltiorrhiza*, Semen Persicae, *P. quinquefolius*, *A. pilosa*, *A. capillaris*, and Eupolyphaga Seu Steleophaga, respectively (Figure 2(b)).

### 3.2. Chemical Composition of STRGD

**Figure 3:**
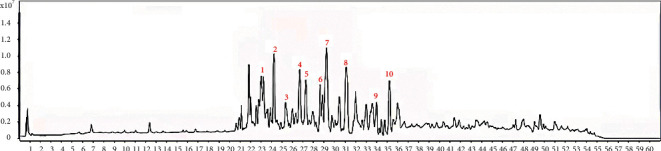
Alcohol extractions of STRGD were qualitatively analyzed with HPLC. The numbers in the chromatograms indicate the constituent peaks. 1. Tanshinone IIB. 2. Chikusetsu saponin Iva. 3. Agrimonolide. 4. Danshenxinkun A. 5. Dihydrotanshinone (I). 6. Ginsenoside Rg2. 7. Ginsenoside Rg3. 8. Cryptotanshinone. 9. Isotanshinone IIA. 10. tanshinone IIA. A typical chromatogram is shown (*n* = 3).

HPLC identified the following 10 main chemical compounds in STRGD: tanshinone IIB, chikusetsu saponin IVa, agrimonolide, danshenxinkun A, dihydrotanshinone I, ginsenoside Rg2, ginsenoside Rg3, cryptotanshinone, isotanshinone IIA, and tanshinone IIA (Figure 3).

### 3.3. Acquisition of Targets Associated with LIHC

**Figure 4:**
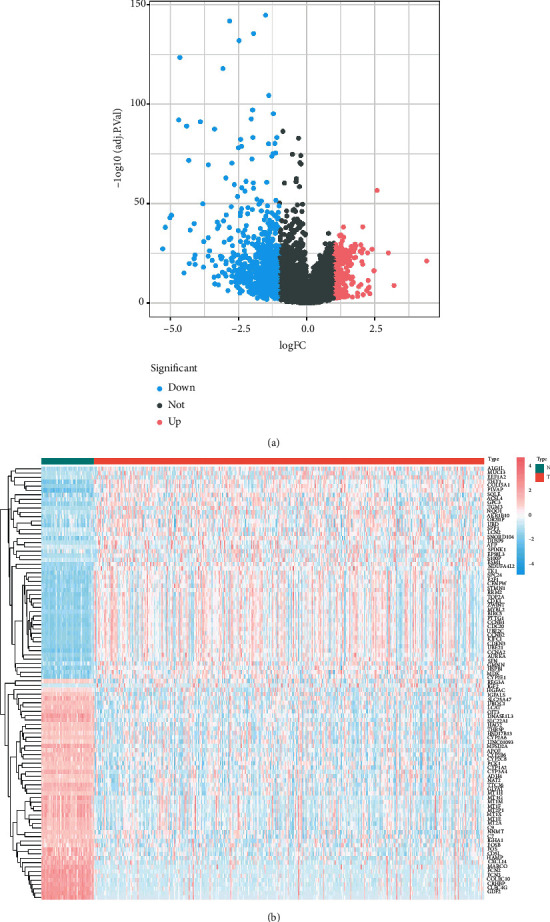
Differentially expressed genes identified in the TCGA database. (a) Volcano map and (b) hierarchical clustering heat map of differentially expressed genes between LIHC and control.

**Figure 5:**
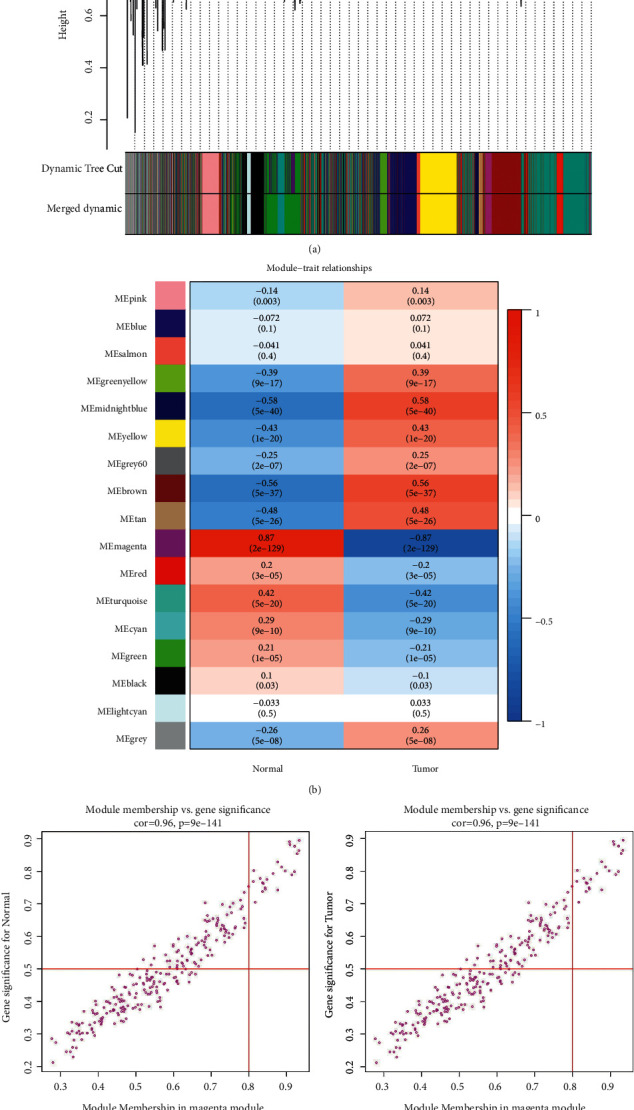
WGCNA analysis. (a) Clustering dendrogram (top) and gene modules with different colors (bottom). (b) Correlation heatmap of modules and clinical traits. (c) Gene correlation scatter plots of the MEmagenta model.

An LIHC whole gene expression data set was downloaded from TCGA. The project ID was TCGA-LIHC, the workflow type was HTSeq-FPKM, and the data type was gene expression quantification. A total of 424 samples were obtained, comprising 374 and 50 LIHC and normal tissues, respectively. Clinical data were obtained for 365 cases; samples with missing clinical data were excluded from the analysis. Through differential analysis of the quality-screened data set, 1095 DEGs were obtained, including 271 upregulated and 824 downregulated genes, which are displayed in a volcano map and heatmap in Figures 4(a) and 4(b). A gene coexpression network of TCGA-LIHC was constructed using the WGCNA *R* package, and the optimal soft threshold power *β* was determined to be 6. The dynamic tree cut method and average hierarchical clustering generated 17 modules (Figure 5(a)). Analysis of the modules and traits using a heatmap of module-trait relationships showed that the MEmagenta module had the closest correlation with LIHC (*R* = 0.87, *P* < 0.01; Figures 5(b) and 5(c)). Furthermore, a total of 253 coexpressed genes were found in the ME magenta module. Considering the intersection of coexpressed genes and DEGs, 165 genes were identified as LIHC disease targets (Figure 2(c)). Considering the intersection of disease targets and compound targets of STRGD, we found 24 drug-disease targets, which were considered tumor targets of STRGD (Figure 2(d)).

### 3.4. Construction of a Drug-Compound-Target-Disease Network

**Figure 6:**
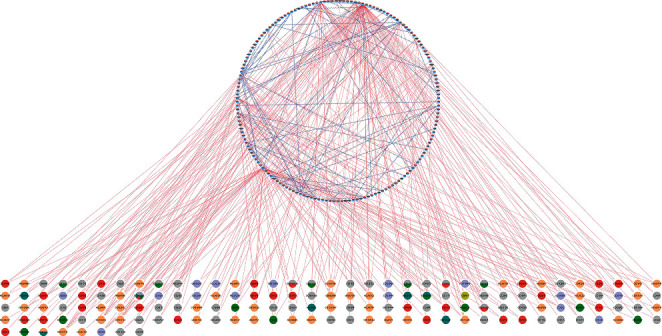
The drug-compound-target-disease network of STRGD for the treatment of LIHC. Nodes represent compounds and targets. The node below indicates the 148 active compounds and the colors represent different component sources. The node enclosed in a circle indicates 165 LIHC disease targets, in which blue is the co-expressed gene obtained from WGCNA and red is DEGS. The red edges represent the interaction between the compounds and the targets; the blue edges symbolize the interaction between the targets.

Based on the tumor targets of STRGD and disease targets identified, STRING and Cytoscape 3.8.0 were used to construct a drug-compound-target-disease network, which was composed of 313 nodes (148 bioactive compounds and 165 disease targets) and 354 edges (Figure 6). The network showed that some active compounds of STRGD were present in multiple Chinese medicines. Furthermore, a single compound could correspond to multiple targets, and a single target could be regulated by multiple compounds. The regulatory network of STRGD was a complex system, with multiple compounds and multiple targets acting together.

### 3.5. Survival Analysis

**Figure 7:**
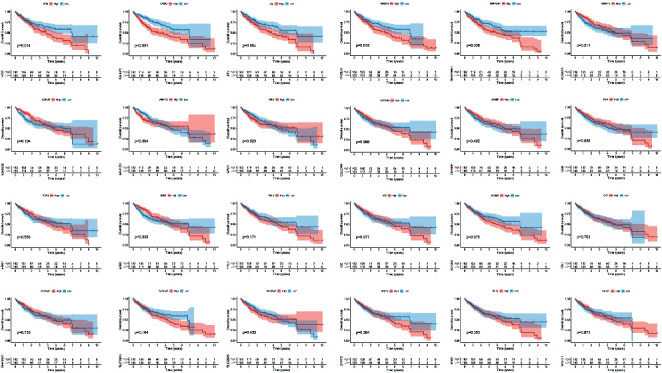
Identification of key genes affecting overall survival of LIHC from tumor targets of STRGD. According to the median expression level, patients are divided into high-level and low-level groups.

Kaplan–Meier survival curves were constructed to analyze the effects of tumor targets of STRGD on patient survival (Figure 7). The expression levels of 24 genes were found to have specific effects on survival. Insulin-like growth factor-binding protein 3 (IGFBP3), serpin family E member 1 (SERPINE1), fos proto-oncogene (FOS), phosphoglycerate dehydrogenase (PHGDH), and jun proto-oncogene (JUN) significantly affected the survival of LIHC patients (*P* < 0.05) and were correlated with low survival rates.

### 3.6. Biological Functional Analysis

**Figure 8:**
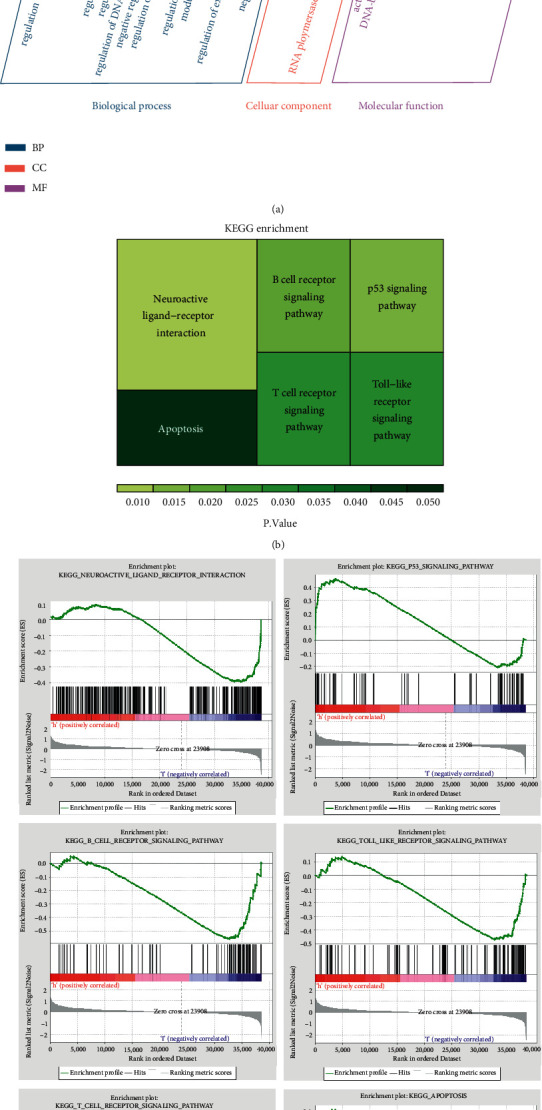
Enrichment analysis of STRGD against LIHC. (a) GO enrichment analysis. (b) KEGG pathway enrichment analysis. (c) Enriched KEGG pathways predicted by GSEA analysis. The same GSEA results as KEGG analysis are shown.

GO and KEGG enrichment analyses of the tumor targets of STRGD were conducted using *R*. GO terms with high enrichment scores were mainly associated with transcription factor binding; protein kinase regulator, inhibitor, and activator activities; regulation of signaling receptor activity; receptor ligand activity; regulation of B cell differentiation; negative regulation of B cell activation; and RNA polymerase II transcription regulator complex (Figure 8(a)). The KEGG pathway enrichment analysis results showed that STRGD may exert anticancer effects through actions, such as neuroactive ligand-receptor interaction; P53, B-cell receptor, Toll-like receptor, and T-cell receptor signaling pathways; and apoptosis (Figure 8(b)). GSEA corroborated the involvement of the above mentioned pathways. These findings indicated that STRGD exert its effects in the treatment of LIHC by directly regulating these pathways through the targets (Figure 8(c)).

### 3.7. Immune Infiltration Analysis

**Figure 9:**
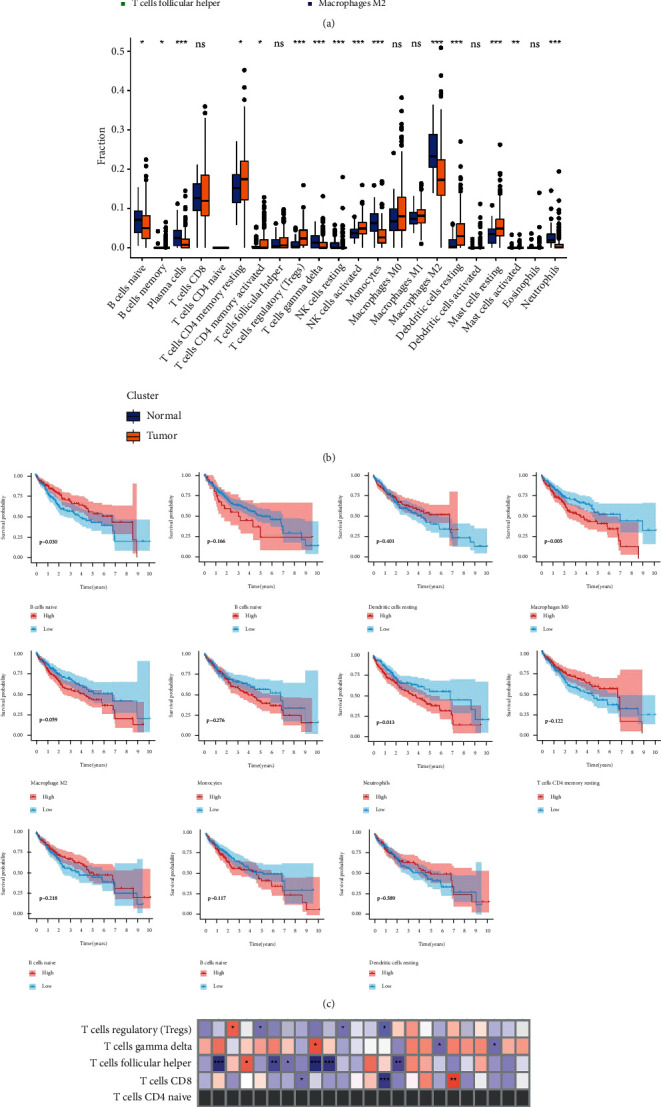
Immune cell infiltration analysis in TCGA-LIHC cohort. (a, b) Bar chart and box plots of 22 immune cell proportions between LIHC and normal control tissues. (c) Kaplan–Meier curves related to the differentially distributed immune cells. (d) Correlation matrix between immune infiltration level and genes related to survival and prognosis from tumor targets of STRGD. Red/blue color indicates positive/negative correlation. The correlation index between genes expression and cells infiltration is reflected by the depth of the color.

A bar chart and box plot of the proportions of immune cells in LIHC and normal tissues were constructed using *R* (Figures 9(a) and 9(b)). The results indicated that naïve B cells, plasma cells, resting and activated memory CD4+ T cells, regulatory T cells (Tregs), *γδ*T cells, activated and resting natural killer cells, monocytes, M2 macrophages, resting dendritic cells (DCs), resting and activated mast cells, and neutrophils showed statistically significant differences in their degree of infiltration between LIHC and normal tissues.

Kaplan–Meier survival curves and log-rank tests were used to evaluate the correlation between the infiltration degree of each immune cell type and prognosis (Figure 9(c)). The results showed that naïve B cells, resting memory CD4+ T cells, CD8+ T cells, follicular helper T cells, Tregs, M0 macrophages, M2 macrophages, monocytes, activated and resting DCs, and neutrophils were significantly related to the prognosis of LIHC.

Spearman correlation analysis of the correlation between the expression of tumor targets of STRGD and the infiltrating immune cells is illustrated in Figure 9(d). The results showed that the immune cells that were highly, significantly associated with high expression of drug-disease target genes were T-and B-cell subsets, such as naïve B cells, activated and resting memory CD4+ T cells, and Tregs. These results were consistent with those of GSEA and KEGG analysis of these genes, highlighting the roles of T- and B-cell subsets in mediating the therapeutic effects of STRGD.

### 3.8. Molecular Docking

**Figure 10:**
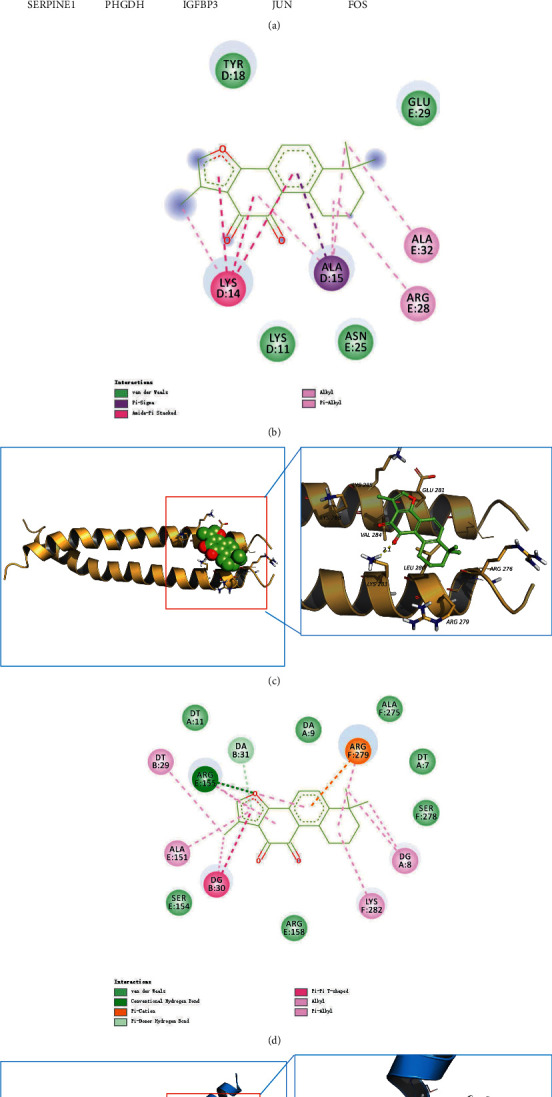
Results of molecular docking. (a) Heat map of molecular docking results. (b, c) Binding poses of tanshinone IIA at the binding site of 1JUN and 2D interaction diagrams. (d, e) Binding poses of tanshinone IIA at the binding site of 1FOS and 2D interaction diagrams.

Molecular docking was conducted to calculate the binding energy between major chemical compounds of STRGD (azelaic acid, *β*-sitosterol, *γ*-sitosterol, hexadecanoic acid, kaempferol, miltirone, neocryptotanshinone_Ii, progesterone, quercetin, rhein, serotonin, and tanshinone IIA) and the core target proteins related to prognosis (IGFBP3, SERPINE1, FOS, PHGDH, and JUN). The batch molecular docking results are shown in a heatmap in Figure 10(a). The results indicated that nearly all the active ingredients involved in the docking had specific regulatory effects on the target proteins. This observation corroborated that the regulatory STRGD network is a complex system with multiple compounds and targets.

### 3.9. MDS and MM-PBSA Calculations

**Figure 11:**
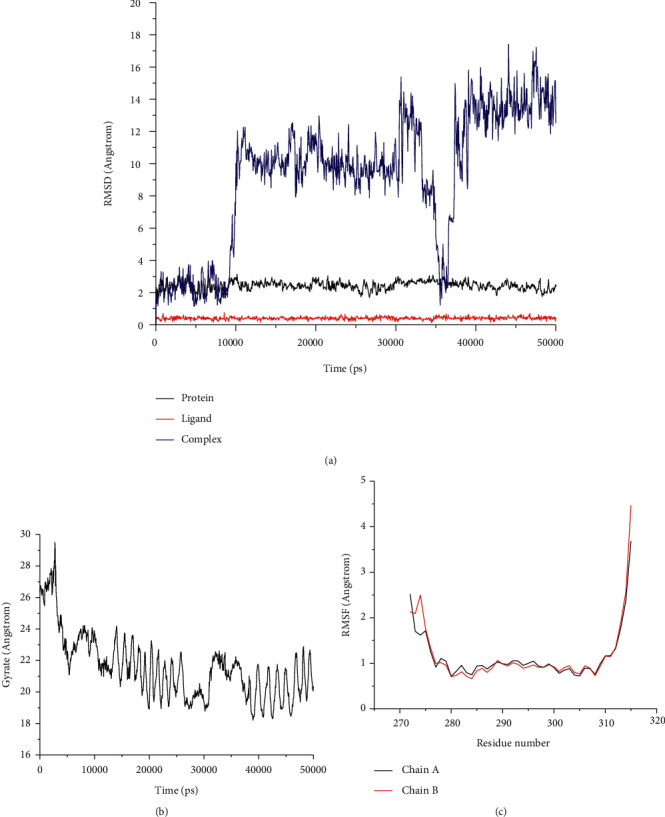
Analysis of MD trajectories generated by Gromacs for 1JUN-tanshinone IIA. (a) RMSD of protein and tanshinone IIA. (b) Gyrate. (c) RMSF in residues of proteins.

**Figure 12:**
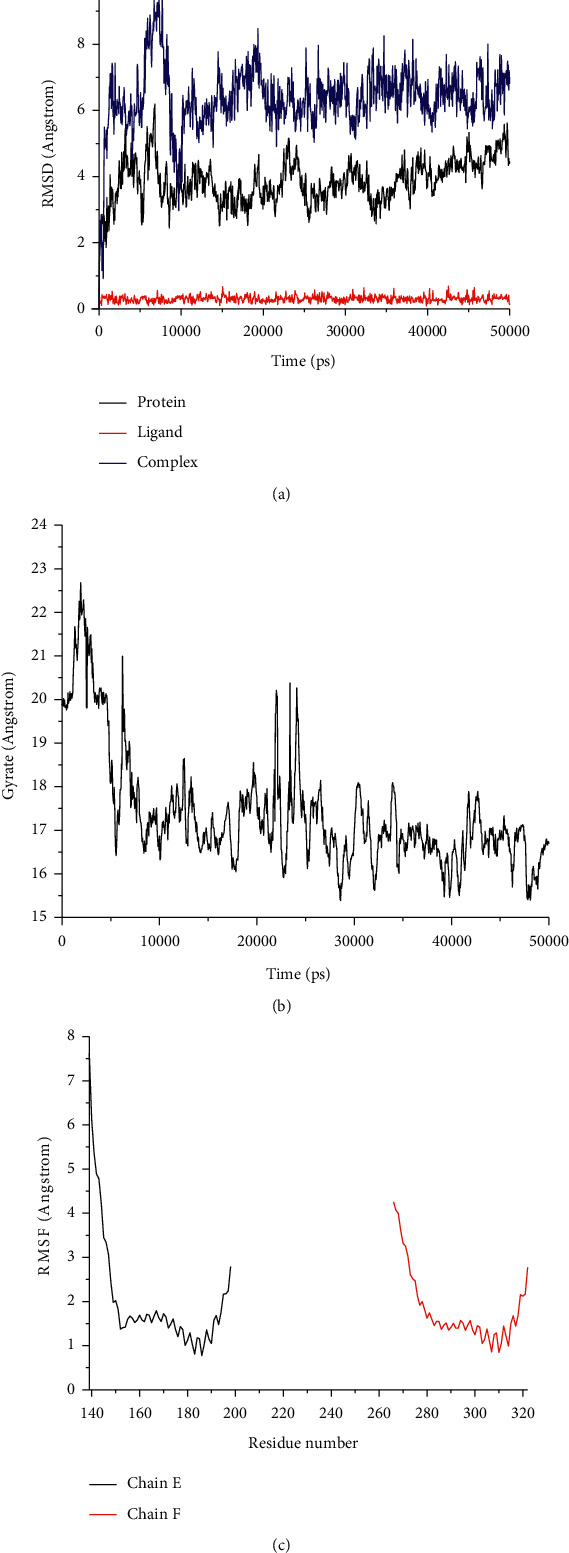
Analysis of MD trajectories generated by Gromacs for 1FOS-tanshinone IIA. (a) RMSD of protein and tanshinone IIA. (b) Gyrate. (c) RMSF in residues of proteins.

**Figure 13:**
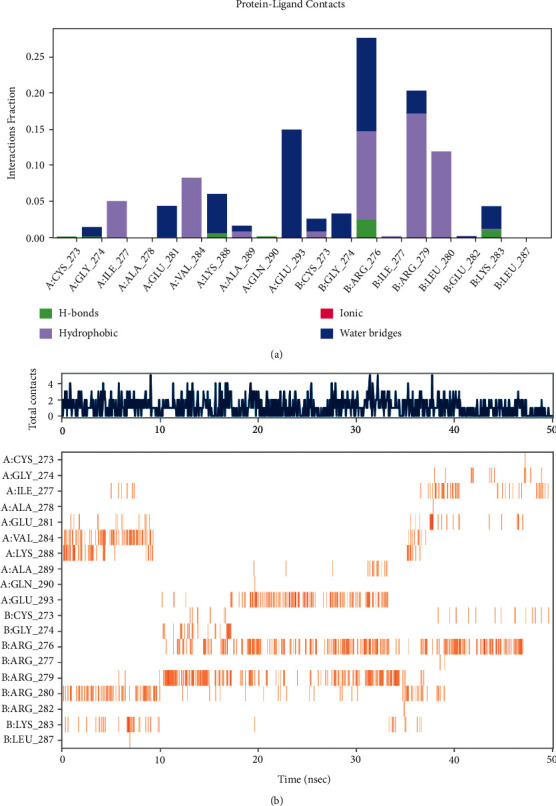
Nonbonded interactions between the molecules and the residues at the binding site for 1JUN-tanshinone IIA. (a) Timeline representation of the interactions and contacts (H-bonds, Hydrophobic, Ionic, Water bridges). (b) Top panel shows the total number of specific contacts the protein makes with the ligand over the course of the trajectory. The bottom panel shows which residues interact with the ligand in each trajectory frame. Some residues make more than one specific contact with the ligand, which is represented by a darker shade of orange, according to the scale to the right of the plot.

**Figure 14:**
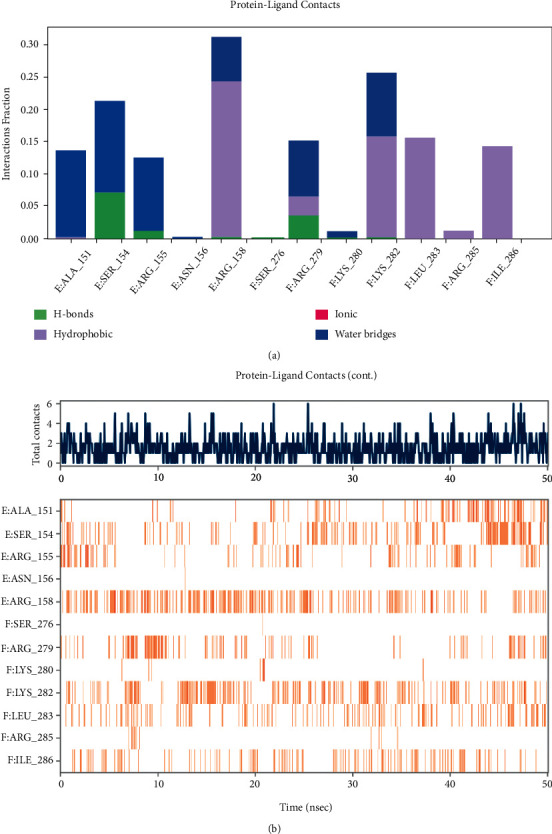
Nonbonded interactions between the molecules and the residues at the binding site for 1FOS-tanshinone IIA. (a) Timeline representation of the interactions and contacts (H-bonds, Hydrophobic, Ionic, Water bridges). (b) Top panel shows the total number of specific contacts the protein makes with the ligand over the course of the trajectory. The bottom panel shows which residues interact with the ligand in each trajectory frame. Some residues make more than one specific contact with the ligand, which is represented by a darker shade of orange, according to the scale to the right of the plot.

Root-mean-square deviation (RMSD) analysis of protein atoms showed that the 1FOS complex with tanshinone IIA had an average RMSD value of 0.64 nm (Figure 11(a)). However, the 1JUN complex with tanshinone IIA had a higher average RMSD value of 0.93, suggesting it is less stable than the former complex (Figure 12(a)). Moreover, these results provided evidence supporting the docking scores. It is also important to measure the RMSD of ligand atoms. For the 1FOS and 1JUN complexes with ligands, tanshinone IIA had similar deviations with average RMSD values of 0.30 and 0.41, respectively, suggesting improved stabilization in both cases (Figures 11(a) and 12(a)). Generally, the gyrate of 1FOS and 1JUN protein-ligand complexes showed a downward trend. The complexes of 1FOS and 1JUN with tanshinone IIA had average gyrate values of 17.38 Å and 21.55 Å (Figures 11(b) and 12(b)), respectively. Root-mean-square fluctuation (RMSF) is a good measure of binding site adaptation and other phenomena. For complexes of 1FOS and 1JUN with tanshinone IIA, the fluctuations in a cluster of binding site residues were minimal. The minor fluctuations at the binding site residues were nearly similar for both complexes (Figures 11(c) and 12(c)). The results of protein-ligand contact analysis indicated that tanshinone IIA produced hydrophobic and water bridges with many key residues at the binding site (Figures 13 and 14). As for the MDS of 1FOS protein-ligand complexes, the MM-PBSA results showed that tanshinone IIA had van der Waal and binding free energy values of −28.19 and −36.09 kJ/mol, respectively, whereas the corresponding results for 1JUN protein-ligand complexes were −22.34 and −27.88 kJ/mol, respectively.

## 4. Discussion

In this study, transcriptomics, Kaplan–Meier analysis, HPLC, molecular docking studies, MDSs, immune infiltration analysis, and bioinformatics analyses were conducted to systematically explore the relationships between STRGD compounds and survival prognosis as well as immunity in patients with LIHC.

**Figure 15:**
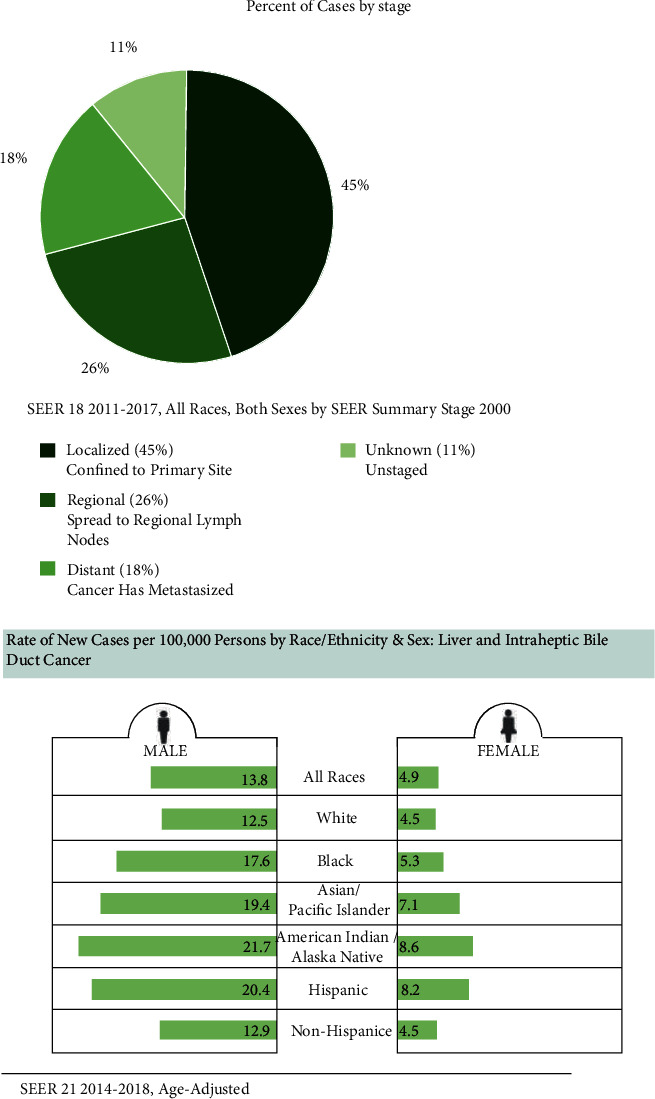
Characteristics of LIHC cohort from the SEER database.

Initially, we compared clinical data retrieved from the TCGA-LIHC data set with those from the SEER database and found that they were similar (Figure 15). Most cases involved male patients and patients diagnosed early, demonstrating the relative reliability of the TCGA-LIHC dataset.

The database search identified 148 bioactive compounds and 2045 drug targets for the eight Chinese medicines that serve as the base materials of STRGD preparation. The most significant LIHC-related gene modules were identified using WGCNA, which revealed that genes in the MEmagenta module were the most significantly related to LIHC. At the intersection with DEGs, 165 genes were found, which were LIHC disease targets. There were 24 shared genes between drug and disease targets. Kaplan–Meier analysis and log-rank tests showed that five genes, i.e., IGFBP3, SERPINE1, FOS, PHGDH, and JUN, were significantly associated with the survival of LIHC patients. Previous studies have indicated that SERPINE1 and FOS can serve as predictive markers of overall survival [42, 43] and that IGFBP3 is also related to survival [44].

GSEA of LIHC and normal tissues suggested specific biological and pharmacological mechanisms underlying LIHC development and STRGD action, respectively. GSEA revealed similar signaling pathways as KEGG enrichment analysis, such as neuroactive ligand-receptor interaction; P53, B-cell receptor, Toll-like receptor, and T-cell receptor signaling pathways; and apoptosis. A previous study suggested that annexins played a role in LIHC carcinogenesis and development through the neuroactive ligand-receptor interaction pathway [45]. High expression of CCNB1, RRM2, and CDK1 enriched in the P53 signaling pathway has been correlated with poor survival [46]. The P53 Pro/Pro and MDM2 G/G genotypes may be involved in the occurrence and progression of LIHC and are prognostic risk markers for LIHC with malignant characteristics [47]. CD2+B cells, CD27^−^isotypic conversion memory B cells, and primitive B cells have been identified as prognostic determinants of survival and are associated with higher survival rates [48]. Engineered T cells can be used in combination with cancer vaccines and immune checkpoint blockers to improve the efficacy of LIHC immunotherapy [49]. Emodin extracted from *Radix rhei Et Rhizome* may induce apoptosis of LIHC cells through apoptosis-related pathways [50]. Our study indicates that the therapeutic mechanisms of STRGD may be mediated through the direct regulation of targets, affecting the identified key pathways with high LIHC-related significance.

The tumor microenvironment, including both innate and adaptive immune cells, is a target of immunotherapy approaches and affects the survival and prognosis of patients with LIHC [51]. The expression levels of the tumor targets of STRGD were found to be closely related to the levels of immune cell infiltration. Kaplan–Meier analysis and log-rank tests showed that T- and B-cell immune subsets, such as naïve B cells, resting memory CD4+ T cells, and Tregs, were also positively associated with the survival of LIHC patients. Tregs may be an important immunosuppressive target in LIHC [52]. B- and T-cell infiltration may be related to good prognosis of immune-high LIHC [51], whereas CD4+ T cell overexpression may inhibit the occurrence of LIHC [53]. We found that STRGD promotes tumor immunity by modulating multiple immune cell populations, thus improving the survival and prognosis of LIHC patients.

The results of the enrichment and immune infiltration analyses indicated that the therapeutic effect of STRGD on LIHC involves the P53 and apoptosis pathways and is closely related to T and B cells. Survival analysis based on drug-disease intersection targets showed that STRGD regulates targets such as JUN and FOS to promote the survival of LIHC patients. The T- and B-cell receptor signaling pathways identified by GSEA show that both are related to activator protein 1 (AP1). AP1 is a dimeric transcription factor composed of JUN, FOS, ATF, and MAF proteins. FOS protein is often dimerized with JUN proteins to form AP1. Treg overexpression may promote LIHC progression by activating AP1 and inhibiting dendritic cell-mediated immunity [51]. STRGD has been reported to reduce the expression of Tregs and restore immune function [22], which is consistent with our preliminary results.

Molecular docking results showed that tanshinone IIA had good binding activity with IGFBP3, SERPINE1, FOS, PHGDH, and JUN. This indicates that tanshinone IIA may be the main active compound of STRGD in LIHC treatment, which is consistent with the HPLC results. To investigate the effect of tanshinone IIA binding to AP1, we visualized the target proteins 1FOS and 1JUN docked with tanshinone IIA (Figures 10(b)–10(e)). Furthermore, MDS and post MDS analysis were conducted for the equilibrated protein-ligand complexes. MDS provides more accurate binding patterns of ligands than molecular docking does, eliminating the limitations of docking studies and providing an in-depth understanding of binding free energy and binding affinity [54]. Parameters such as RMSD and RMSF for ligands and proteins are indicative of the stability of the system during the MDS timescale [55]. The strength and binding affinity of the complexes depends on nonbonded interactions, such as hydrogen bond and ionic and hydrophobic interactions [56]. Gyrate provides information on the relationship between proteins and ligands during MDS [57]. MM-PBSA calculations were used to estimate the binding free energies of the post-MDS trajectories [58]. The results indicated that tanshinone IIA forms a stable complex with 1FOS and 1JUN proteins and could potentially inhibit these proteins. Thus, the docking studies and MDS indicated that the potential LIHC-immune-related targets 1FOS and 1JUN bind firmly with tanshinone IIA.

## 5. Conclusion

The mechanisms underlying the improvement in the immune and survival status of LIHC patients by STRGD include interactions of STRGD compounds with drug-disease cross targets and regulation of gene expression and tumor immunity. Computational evaluation and network pharmacology methods can be used in further studies on the potential usefulness of tanshinone IIA, identified as a lead compound of STRGD in this study, in the development of drugs targeting 1JUN and 1FOS for the treatment of LIHC.

## Data Availability

All data generated or analyzed during this study are included in this published article and its supplementary information files.

## Abbreviations

LIHC: Liver hepatocellular carcinoma
STRGD: Shentao Ruangan decoction
HPLC: High-performance liquid chromatography
MDS: Molecular dynamics simulation
TCMSP: Traditional Chinese Medicine Systems Pharmacology Database and Analysis Platform
DEGs: Differentially expressed genes
TCGA: The Cancer Genome Atlas
WGCNA: Weighted gene coexpression network analysis
ICC: Intrahepatic cholangiocarcinoma
TACE: Transcatheter arterial chemoembolization
TCM: Traditional Chinese Medicine
IGFBP3: Insulin-like growth factor-binding protein 3
SERPINE 1: Serpin family E member 1
FOS: Fos proto-oncogene
PHGDH: Phosphoglycerate dehydrogenase
JUN: Jun proto-oncogene
PPI: Protein-protein interaction
DCs: Dendritic cells
GSEA: Gene set enrichment analysis
KEGG: Kyoto Encyclopedia of Genes and Genomes
Tregs: Regulatory T cells
GO: Gene ontology
MM-PBSA: Molecular Mechanics Poisson–Boltzmann Surface Area
RMSD: Root mean square deviation
RMSF: Root mean square fluctuation
AP1: Activator protein 1.

## Supplementary Materials

Supplementary Materials S1 File. The data contained in this file are all the data ultimately used for the analysis in this article. File name: Sheet 1. Title of data: clinical data retrieved from the TCGA-LIHC project. Description of data: overall survival time, survival status, and other clinical information of liver cancer were retrieved from TCGA database. File name: Sheet 2. Title of data: the details of the MDS and MM-PBSA calculations. Description of data: the protocol of the MDS and MM-PBSA calculations.
