# Supplementary material for: Mechanism of a Herbal Formula Associated with Prognosis and Immune Infiltration in LIHC: Transcriptomics Analysis and Molecular Dynamics Simulations
Source: Evid Based Complement Alternat Med. 2022 Jun 15;2022:6084321. doi: 10.1155/2022/6084321 (PMC9217603; doi:10.1155/2022/6084321)
Supplement: Supplementary Materials — S1 File. The data contained in this file are all the data ultimately used for the analysis in this article. File name: Sheet 1. Title of data: clinical data retrieved from the TCGA-LIHC project. Description of data: overall survival time, survival status, and other clinical information of liver cancer were retrieved from TCGA database. File name: Sheet 2. Title of data: the details of the MDS and MM-PBSA calculations. Description of data: the protocol of the MDS and MM-PBSA calculations. [file 6084321.f1.zip › 6084321.f1/sheet2.docx]

**Molecular Dynamics Simulations**

All molecular dynamics (MD) simulations were run only on the ligand and receptor using Gromacs 5.14 software. The complexes were first solvated with SPC water molecules and positioned in the center of a cubic box. The solvated complexes were then subjected to energy minimization for 5000 steps using a combination of steepest descent and conjugate gradient algorithms. The minimized systems were afterward smoothly heated from 0 to 300 K over 50 ps with a weak restraint of 10 kcal/mol/Å on the receptor. Further MD simulations were executed for 1000 ps to equilibrate the investigated complexes. Eventually, MD simulations for each complex were conducted for 50 ns. In MD simulations, the Particle Mesh Ewald (PME) method was applied for treating the long-range electrostatic interactions under periodic conditions with a direct space cutoff of 12 Å. In addition, the linear constraint solver (LINCS) algorithm was applied for covalent bond constraints. In order to maintain the temperature at 300 K, Langevin dynamics with collision frequency gamma_ln set to 1.0 was applied. The pressure was controlled using a Berendsen barostat with a relaxation time of 2 ps. Bonds involving hydrogen atoms were also constrained via a SHAKE algorithm with a time step of 2 fs. All MD simulations were carried out using a CPU version of gmx_mdrun tool, implemented in gromacs software. The pymol software was employed for 3D and 2D visualizations of the complex interactions.

**Binding free-energy calculation.** The binding free energies for complexes were calculated with g_mmpbsa software in GROMACS5.14 based on the trajectory approach using MD trajectory for each complex. The binding free energy was figured out using following equations:

The complex binding free-energy of MM/PBSA is defined as

△G_bind_=G_complex_-G_free-prrotein_-G_free-ligand_

In a solution, free energy of the molecules can be expressed as

G=E_gas_-TS_gas_+G_solvate_

G_solvate_=G_polar_+G_apolar_

Molecular mechanics potential energy, E_MM_, includes the energy of both bonded and non-bonded. It is calculated based on this equation,

E_gas_=E_MM_=E_bond_+E_angle_+E_dihedral_+E_vdw_+E_coulomb_

Where, E_bond_ E_angle_ and E_dihedral_ are bonded interactions consisting of bond, angle, dihedral. The non-boned interactions include both van der Waals (E_vdw_) and Coulomb charge effect (E_coulomb_)

G_polar_=G_PB_

G_apolar_=G_surface_=γA+b

The solvability of MM/PBSA is consisted of polar (G_polar_) and apolar (G_apolar_). G_polar_ is calculated by solving the Poisson-Boltzmann (PB) equation; G_apolar_ is estimated according to the surface area of experience method, in this, γ is a coefficient related to surface tension of the solvent, A is solvent accessible surface area (SASA) and b is fitting parameter.
